# Repeatability of the Maximum Standard Uptake Value (SUVmax) in FDG PET

**DOI:** 10.4274/Mirt.76376

**Published:** 2014-02-05

**Authors:** Henry Lindholm, Johan Staaf, Hans Jacobsson, Fredrik Brolin, Robert Hatherly, Alejandro Sânchez-Crespo

**Affiliations:** 1 Karolinska University Hospital, Department of Radiology, Stockholm, Sweden; 2 Karolinska University Hospital, Department of Hospital Physics, Stockholm, Sweden

**Keywords:** Examinations and diagnoses, Positron-emission tomography, Fluorodeoxyglucose F18, reproducibility of results

## Abstract

**Objective**: SUV_max_ is often calculated at FDG PET examinations in systematic studies as well as at clinical examinations. Since SUV_max_ represents a very small portion of a lesion it may be questioned how statistically reliable the figure is. This was studied by assessing the repeatability of SUV_max_ between two FDG acquisitions acquired immediately upon each other in patients with chest lesions.

**Methods**: In 100 clinical patients with a known chest lesion, two identical 3 min PET registrations (PET1 and PET2, respectively) were initiated within 224±31 sec of each other. The difference in SUV_max_ between the lesion for the two PET scans (ΔSUVmax) was calculated and the uncertainty expressed as the coefficient of variation, CV (%). The correlation between ΔSUV_max_ and the lowest SUV_max_ from PET1 or PET2, the approximate metabolic lesion volume, the time from FDG injection to PET1 and the time between PET1 and PET2, respectively, was also assessed.

**Results**: In 56 patients SUV_max_ increased at the second acquisition and in 44 patients it decreased. Mean of SUV_max_ was 7.8±6.1 and 7.8±6.2 for PET1 and PET2, respectively. The mean percentage difference was 0.9±7.8. The difference was not significant (p=0.20). CV gave an uncertainty of 4.3% between the two measurements which is a strong indicator of equivalence. There was no correlation between ΔSUV_max_ and any of the assessed four parameters. The difference between the acquisitions, 0.9%, was much lower compared to the 3 previous published similar, but more restricted studies where the difference was 2.5-8.2%.

**Conclusion**: From camera and computational perspectives, SUV_max_ is a stable parameter

**Conflict of interest:**None declared.

## Abstract (tr)

**Amaç**: Sistematik çalışmalarda ya da klinikte yapılan muayenelerde yapılan FDG PET çalışmalarında SUV_max_ sıklıkla hesaplanmaktadır. SUV_max_ bir lezyonun küçük bir parçasını temsil etttiği için, bu rakamın istatstiksel olarak güvenilirliği sorgulanabilir. Bu amaçla toraks lezyonu olan hastalarda derhal ardarda yapılan iki FDG PET çekimlerinde SUV_max_’ın tekrarlanabilirliği araştırılmıştır.

**Yöntem**: Bilinen toraks lezyonu olan 100 hastada, iki eşdeğer 3 dakikalık PET çekimi (sırasıyla, PET1 ve PET2) aralarında 224±31 saniye olacak şekilde gerçekleştirildi. İki PET taraması arasında lezyondaki SUV_max_ farkı (ΔSUV_max_) hesaplandı ve belirsizlik varyasyon katsayısı (CV (%)) olarak ifade edildi. ΔSUV_max_ ile; PET1 veya PET2 deki en düşük SUV_max_ değeri, yaklaşık metabolik lezyon hacmi, FDG enjeksiyonundan PET1 e kadar geçen zaman ve PET1 ve PET2 arasındaki süre arasındaki korelasyon hesaplandı.

**Bulgular**: İkinci çekimde, 56 hastada SUV_max_ arttı, 44 hastada azaldı. Ortalama SUV_max_ PET1’de 7.8±6.1, PET2’de 7.8±6.2 idi. Ortalama yüzde fark 0.9±7.8 idi. Fark önemli bulunmadı (p=0.20). İki ölçüm arasında CV’nin verdiği belirsizlik %4.3 idi ve eşdeğerliğin kuvvetli bir göstergesiydi. ΔSUV_max_ ile diğer araştırılan dört parametre arasında korelasyon görülmedi. %0.9 olarak bulunan iki çekim arası fark değeri, daha önce yayınlanan 3 benzer ancak daha kısıtlı çalışmalarda bulunan farklara (%2.5-8.2) göre daha düşüktü.

**Sonuç**: Kamera ve hesaplama açısından SUV_max_ stabil bir parametredir.

## INTRODUCTION

In medical imaging there is a tendency to accord numbers special value, irrespective of how robust they are (1). Positron Emission Tomography (PET) represents such a quantitative process, as the raw signal can be transformed into absolute concentrations of the radiotracer after a series of transitions and corrections. Hence, PET is often used for quantification of different molecular processes. This is usually made by calculating the Standardized Uptake Value (SUV). In a given image volume, the SUV is defined as the tracer concentration, normalised to the administered activity and e. g. the body weight. The mean or the maximum SUV (SUV_mean_ and SUV_max_, respectively) within a somehow defined Volume-Of-Interest (VOI), are commonly used figures for description of the tracer uptake.

SUV based PET image quantifications are hampered by technical, physical and biological processes. Despite these well known limitations, SUV calculations are commonly used at clinical examinations as well as at scientific studies. In this respect, SUV quantifications are often ascribed a value which is overrated in relation to their actual precision (1,2,3,4).

SUV_max_ is regarded as a figure of merit for prognosis and therapy evaluation (5,6,7,8). While SUV_mean_ heavily depends on the definition of the VOI, SUV_max_ does not, thereby being almost reader independent. Furthermore, SUV_max_ is less affected by partial volume effects while it, on the other hand, is affected by the image noise (9,10). This may be considerable as the administered activity and the scanning time is restricted in clinical practice. In addition, SUV_max_ represents a very small portion of the VOI, why it may be questioned how well it reflects the biology of an entire lesion (11). In the current report, the statistical reliability of SUV_max_ has been studied by assessing its repeatability in an uptake of a chest lesion between two identical acquisitions obtained immediately upon each other in clinical patients.

## MATERIALS AND METHODS

**Patients**

The study is based on 100 clinical patients (mean age 64 years; 53 males and 47 females) with suspected tumours of the chest, referred for a clinical PET/CT examination with [^18^F]-2-fluoro-2-deoxy-D-glucose (FDG). The Regional Research Ethical Committee approved the study.

**Examination**

Approximately one hour after i. v. administration of 4 MBq/kg bw of FDG, the examination was initiated. This was accomplished using a Biograph 64 True Point (Siemens Medical Solutions, Erlangen, Germany) with an axial PET Field-Of-View (FOV) of 21.5 cm. First a low dose CT without contrast medium, from the middle skull to the proximal thigh, for photon attenuation and scatter correction of the PET images was performed. Directly following this, the clinical PET-examination was performed with a 3 min acquisition time for each FOV position and normal tidal breathing. Immediately after this, the two additional study-specific identical PET registrations were sequentially acquired (PET1 and PET2, respectively) with an acquisition time of 3 min and one single FOV including the known lesion. There was a mean of 224±31 sec between the beginnings of the two acquisitions. Thereafter, a full-dose CT, with or without administration of i.v. contrast medium, was performed at breath-holding at a mean inspiratory level. The patient did not change position versus the camera during the series of acquisitions.

All PET images were reconstructed using the manufacturer ordered subset expectation maximization algorithm (OSEM) with 4 iterations and a matrix image size of 168x168 pixels, a nominal slice thickness of 5 mm and a voxel volume of 0.08 cm^3^. Corrections for photon attenuation, random coincidences and photon scatter were made.

**Evaluation**

Only patients showing FDG-uptake of the lesion were included. In some patients several lesions were identified, but for all patients only one lesion was studied. The lesions assessed were based on the possibility of including a wide spread of lesion sizes. There were 3 lesions of the chest wall, 16 lesions of the mediastinum/lung hili, and 81 pulmonary lesions. 70 were suspected lung/oesofageal tumours, 16 were metastases or lymphoma/leukaemia, and 14 were considered to have benign explanations (inflammation/infection, radiation pneumonitis, Wegeners granulomatosis or Hamptons hump).

Evaluation of PET1 and PET2 was made using the commercial software allowing simultaneous assessments of both studies at an identical position. SUV_max_ was calculated by allocating a VOI enclosing the uptake with some margin and using this for evaluation of both examinations.

**Figure 1:**
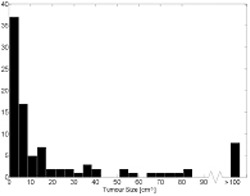
Histogram distribution of the approximately metabolic tumour volume. All tumours larger than 100 cm^3^ are presented as one column.

To estimate the metabolic volume of the lesions, the FDG-uptake was approximated as an ellipsoid/sphere and calculated by manually allocating three orthogonal diameters with a precision of a ½ cm. The distribution of the approximately calculated volumes is shown in Figure 1. The mean volume was 50 cm^3^, and the median volume was 7 cm^3^.

**Data Analysis and Statistics**

The difference between the SUV_max_ of the two PET scans (ΔSUV_max_) was calculated as: ΔSUV_max_ = SUV_max_(PET1) – SUV_max_(PET2)

Since the Jarque-Bera test showed that ΔSUV_max_ was not normally distributed, the Wilcoxon matched-pairs signed-ranks test was used to test whether the two measurements were significantly different. The uncertainty of ΔSUV_max_ (the measurement error) was evaluated according to Dahlberg’s formula and presented as the coefficient of variation, CV (%) (12).

Correlations were assessed between ΔSUV_max_ and the four parameters: the lowest (minimum) of the measured SUV_max_ from either PET1 or PET2, the approximate metabolic lesion volume, the time from FDG injection to PET1, and the time between PET1 and PET2. Analysis showed that this could be made by calculation of Pearson correlation coefficient, which can be used as a measure of strength of linear correlations. Of these, there was a skewed distribution (>1) for the lowest of the measured SUV_max_, the approximate lesion volume and the time from FDG injection to PET1. Thus, a reciprocal transformation was made for these data prior to the analysis. A rule of thumb is that a Pearson correlation coefficient of 0-0.25 indicates little or no relationship (13). The coefficient of determination (R2) was also calculated. This shows the proportion of the total variation explained by the variable studied.

## RESULTS

**Figure 2:**
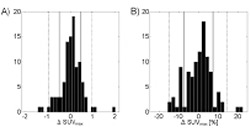
Histogram distribution of the difference ΔSUV_max_=SUV_max_ (PET1)–SUV_max_(PET2). Full and dashed lines represent ±1 and 2 SDs respectively. A, Absolute difference. B, ΔSUV_max_ expressed as a percentage of SUVmax of PET1.

In 56 patients SUV_max_ increased at PET2 and in 44 patients it decreased. Mean of ΔSUV_max_ was 7.8±6.1 and 7.8±6.2 for PET1 and PET2, respectively. The mean percentage difference was 0.9±7.8. The difference was not statistically significant (p=0.20). The distribution of ΔSUV_max_ is shown in Figure 2. Corresponding CV gave a relative uncertainty of 4.3% between the two measurements. The voxel containing the SUV_max_ in the patient with the lowest number of counts after the various transitions and corrections was 28218 which corresponds to a CV of 0.6% (assuming Poisson counting statistics). There was no correlation between ΔSUV_max_ and any of the studied parameters: the lowest of the measured SUV_max_ from either PET1 or PET2, the approximate metabolic lesion volume, the time from FDG injection to PET1, or the time between PET1 and PET2 (Table 1). 

## DISCUSSION

The reproducibility of FDG-PET examinations, i. e. the variation between two different examinations carried out at standardised conditions has been studied by several authors and subjected to a metaanalysis (14). In contrast to this, the repeatability of SUV_max_, i. e. the variability of between two identical consecutive PET scans of the same patient was evaluated in the current study. The aim was to test this at a clinical setting using regularly applied acquisition and reconstruction parameters, thereby also including effects by true image noise. The latter being important as the accuracy of SUV_max_ is limited by a sensitivity to the noise (9,10).

As the lesions were located in the chest, the tracer uptake is affected by respiratory movements. This should not influence the final results, since the movements must have the same impact at the two acquisitions. The metabolic PET volume of the lesion is relevant for the assessments of a possible size influence, why the anatomical (CT) volume was not assessed. The latter would have been much more precise, but of limited value, as the metabolic and anatomic volumes do not always correspond to each other (15). Edge definitions in nuclear medicine examinations, however made, are not precise why the volume assessments are subjected to uncertainties, which represents a limitation of the study. The data presented is dominated by small lesions, this merely reflecting the clinical situation. There was no correlation between the lesion size and ΔSUV_max_ which may have been expected.

Stable conditions between PET1 and PET2 are a prerequisite for the study. This could be influenced by a continuing FDG incorporation still after 60 min, since this has been shown to peak later in tumours (16,17). As there was no difference of SUV_max_ between PET1 and PET2, the (short) interval between the two acquisitions allows for the analyses to be made. This is further supported by the lack of correlation of the time between FDG injection and PET1 versus ΔSUV_max_, as well as the lack of correlation of the time between PET1 and PET2 versus ΔSUV_max_.

The CV corresponding to the lowest number of counts in the study, 0.6%, is much lower than any other uncertainty of the study. Together with the lack of correlation with the lowest of the measured SUV_max_, our findings are hardly influenced by an insufficient number of counts.

There are three previous similar studies (18,19,20). They are not as extensive as in the current report and do not include possible effects on the repeatability by other mechanisms as also studied by us. In the previous studies, the variation of SUV_max_ is much larger than in the current study. In this, there was difference of 0.9% between the two acquisitions, while at the previous studies this figure varied between 2.5-8.2% (Table 2). The activity administered at our examinations, 4 MBq/kg bw, is the lowest compared to the previous studies, why this does not explain our lower value. It should rather have an opposite effect. All previous studies are based on a small number of observations, 8-20 individuals, while we studied a much large cohort to cover differences between patients and lesions sizes. In one of the previous studies, the two comparative acquisitions were initiated as early as 35 min after administration of the radiotracer, why a still ongoing strong increasing tracer uptake may explain the higher uptake at the second acquisition (20). Another study was restricted to normal liver tissue uptake of FDG (18). In contrast to this, the current study is based on pathological lesions located in the chest, which compared to the normal liver uptake are subjected to a lower influence from the surrounding activity, thereby reducing any errors caused by partial volume effects. The divergence towards the previous studies cannot, however, be completely explained other than the current study is based on a larger number of observations and very strictly controlled.

The calculated coefficient of variation (CV), of 4.3% between the examinations is very small. Results of < 5% are considered as a strong indicator of equivalence (12). Consequently, the observed relative measurement error is most likely only due to quantum statistical fluctuations in the disintegration and detection of the positrons. Any odd SUV_max_ results cannot be blamed on a random error in image reconstruction or hardware, but that the value is stable in regards to those factors. 

## CONCLUSION

From camera and computational perspectives, the SUV_max_ is a stable parameter. Any fluctuation can be explained by physiological variations in the radiopharmaceutical uptake, which is what it is meant to describe in the end.

**Acknowledgment**: The authors wish to thank Elisabeth Berg, B. Sc., Karolinska institutet, for the professional statistical analysis.

**Conflicts of interest**: None.

## Figures and Tables

**Table 1:**
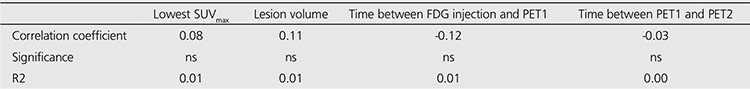
Correlations between ΔSUV_max_ and the lowest of the measured SUV_max_ from either PET1 or PET2, the approximate metabolic lesion volume, the time from FDG injection to PET1 and the time between PET1 and PET2, respectively. ns = not significant

**Table 2:**
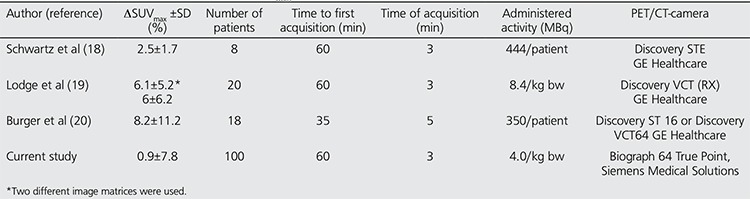
Reports on the repeatability of SUV_max_
